# Lignin Refinery Using Organosolv Process for Nanoporous Carbon Synthesis

**DOI:** 10.3390/molecules25153428

**Published:** 2020-07-28

**Authors:** Imam Prasetyo, Puspita Rahayu Permatasari, William Teja Laksmana, Rochmadi Rochmadi, Won-Chun Oh, Teguh Ariyanto

**Affiliations:** 1 Department of Chemical Engineering, Faculty of Engineering, Universitas Gadjah Mada Jl. Grafika No. 2 Kampus UGM, Yogyakarta 55281, Indonesia; puspitarahayup@gmail.com (P.R.P.); william.teja.l13@gmail.com (W.T.L.); rochmadi@ugm.ac.id (R.R.); 2 The Carbon Material Research Group, Faculty of Engineering, Universitas Gadjah Mada, Yogyakarta 55281, Indonesia; 3 Department of Advanced Materials & Science Engineering, Hanseo University, Chungnam-do 356-706, Korea; wc_oh@hanseo.ac.krn

**Keywords:** carbonization, extraction, organosolv process, porous carbon

## Abstract

Porous carbon has been widely used for many applications e.g., adsorbents, catalysts, catalyst supports, energy storage and gas storage due to its outstanding properties. In this paper, characteristics of porous carbon prepared by carbonization of lignin from various biomasses are presented. Various biomasses, i.e., mangosteen peel, corncob and coconut shell, were processed using ethanol as an organosolv solvent. The obtained lignin was characterized using a Fourier transform infrared (FTIR) spectrophotometer and a viscosimeter to investigate the success of extraction and lignin properties. The results showed that high temperature is favorable for the extraction of lignin using the organosolv process. The FTIR spectra show the success of lignin extraction using the organosolv process because of its similarity to the standard lignin spectra. The carbonization process of lignin was performed at 600 and 850 °C to produce carbon from lignin, as well as to investigate the effect of temperature. A higher pyrolysis temperature will produce a porous carbon with a high specific surface area, but it will lower the yield of the produced carbon. At 850 °C temperature, the highest surface area up to 974 m^2^/g was achieved.

## 1. Introduction

Porous carbon is a versatile material with wide applications, for example: in adsorption [1], separation [2], purification [3], gas storage [4], electrochemical storage [5] and catalysis [6] due to the advantageous properties of pore- and microstructures. There are many precursors for porous carbon preparation, for instance: biomass (natural polymer), synthetic polymer and metal carbide. Biomass materials occur in nature, for example: peat, coconut shell and wood. Porous carbons produced from these natural materials are very dependable concerning elemental composition of materials. These are controlled by e.g., plant age, location and management activities of a plantation. For a synthetic polymer we can adjust the pore structure and purity of materials during synthesis of the polymer [7]. Depending on the preparation method of the polymer, a hierarchical pore architecture and a monomodal pore structure (a narrow pore size distribution) can be obtained [8]. It is important to note that a narrow pore size distribution is important, especially for applications where diffusion limitation is desirable. Metal carbide is new variety of carbon precursor from which high purity and a specific pore range of porous carbon can be obtained [9]. From three types of porous carbon precursors the biomass material is provided in a large amount and is typically cheap. The biomass can be separated into two major products i.e., cellulose and lignin. Lignin has promise as a carbon precursor since it has a large portion of aromatic hydrocarbons [10] and high content of carbon element [11]. Yet, there are still limited studies of lignin for porous carbon synthesis.

To solubilize lignin, a conventional process like the Soda or Kraft process can be employed. In the Soda process, delignification occurs due to a breakdown of ether (-C-O-C-) bonding in lignocellulose. Lignin is then solubilized in an ionic solution. Solubilized lignin can then be precipitated using acids. However, the Soda process also breaks ether bonding in lignin polymeric structures. This produces fragmented lignin polymeric structures with lower polymeric density. The Kraft process is a modification of the Soda process, which uses the addition of sodium sulfide in a sodium hydroxide solvent [12]. Therefore, these two processes are too harsh to solubilize lignin, producing a dramatic change in polymeric structures of lignin. Ionic liquid and organosolv processes are two examples of processes which can solubilize lignin without affecting its original structure. Ionic liquid works due to interference of inter and intramolecular hydrogen bonding in lignocellulose [13]. Ionic liquids include imidazolium, pyridinium, ammonium cations, etc. Despite being able to extract lignin in a high yield, ionic liquid is very expensive [14]. The organosolv process dissolves lignin by breaking lignocellulose in the ether linkage [15]. It employs solvents like dioxane, ethanol, butanol and ethylene glycol. The organosolv solvent is relatively cheap [16]. Furthermore, some studies show promise in extracting lignin using the method [17]. 

We described previously the possibility of preparation of porous carbon from mangosteen peel lignin, which was extracted using an organosolv process at 70 °C [18]. To evaluate the efficacy of the method, it is necessary to expand the study using other lignin sources i.e., corncob and coconut shell, and a higher extraction temperature. This paper presents a comparison of lignin extracted from various biomasses and its use for porous carbon synthesis. To investigate lignin properties and the respective porous carbon produced, characterizations like Fourier transform infrared (FTIR) spectroscopy, scanning electron microscopy (SEM) and N_2_-sorption analysis were employed. The results demonstrate another approach to producing high quality porous carbon by using lignin as a precursor.

## 2. Results and Discussion

### 2.1. Lignin Yield

**Figure 1:**
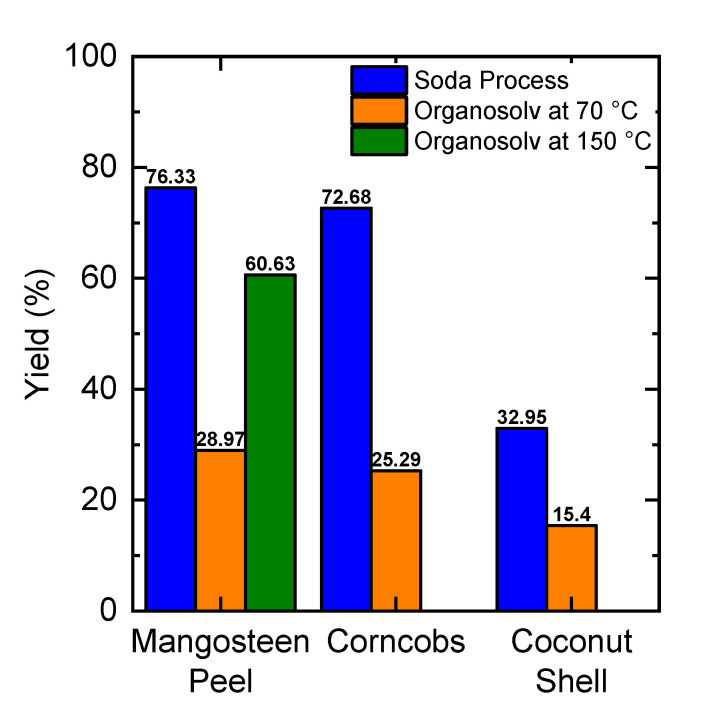
Yield comparison of lignin extracted using organosolv and soda processes. Data of lignin yield of mangosteen peel at 70 °C taken from our previous study [18].

Figure 1 shows the yield of lignin extraction from various biomasses using the organosolv process. Mangosteen peel had a higher yield than the others. To study the influence of temperature, the extraction condition was increased 70 °C to 150 °C. As a representative, mangosteen peel was chosen. When the temperature of the organosolv process was increased from 70 °C to 150 °C, the yield of extraction of mangosteen peel was higher (60.63%). Therefore, increasing temperature can enhance the yield of lignin during organosolv extraction. Ethanol solutions reduce surface tension at high temperatures thereby accelerating diffusion from the breakdown of lignin bonds into organic solutions [19]. As a comparison, all biomasses were also treated using the conventional method of the Soda process (see Figure 1). The solubility of lignin in soda solutions was higher than in organic solutions. The solubility of lignin in a 0.25 M soda solution is 32 g/L [20] while the solubility of lignin in 60% ethanol solution is 16 g/L [21]. 

### 2.2. Characteristics of Lignin

The extraction and isolation processes of lignin have a crucial role in separating lignin from cellulose and hemicellulose without breaking the lignin original molecular structure. The derivations of lignin monomers are *p*-hydroxyphenyl monomers (H type), guaiacyl monomers (G type), and syringyl monomers (S type) [22]. The lignin obtained from every type of biomass waste was analyzed with FTIR spectroscopy to ensure that the lignin was successfully obtained. The graph was then compared with the standard functional group of lignin. 

**Figure 2:**
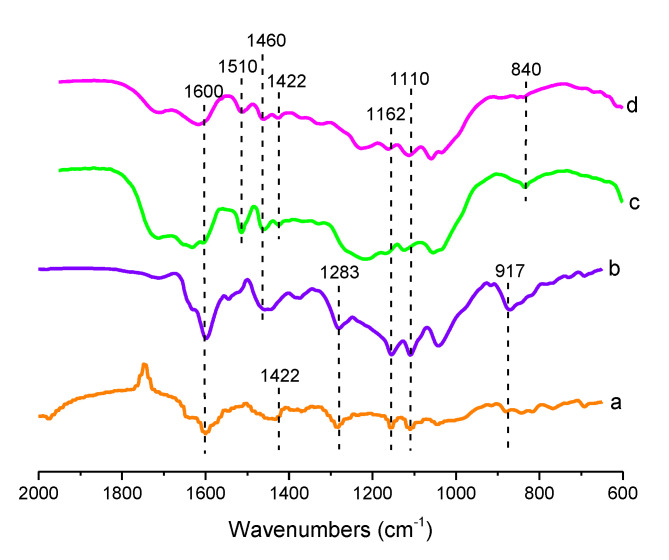
Fourier transform infra-red (FTIR) band from (**a**) LMP, (**b**) LCC, (**c**) LCS, dan (**d**) LMPT. Y-axis is transmittance.

The lignin functional group from the organosolv extractions of various biomass waste can be seen in Figure 2. Lignin from mangosteen peel, lignin from corncob and lignin from coconut shell, which were extracted using the organosolv process at 70 °C, are labeled as LMP, LCC, and LCS, respectively. Additionally, lignin from mangosteen peel, which was extracted using the organosolv process at a higher temperature of 150 °C, is labeled as LMPT. For the spectra evaluation, several publications studying FTIR characterization of lignin were used [22,23,24,25]. At a wavelength of 1600 cm^−1^, lignin in (a), (b), (c), and (d) showed C=C stretching of the aromatic ring in lignin while in 1510 cm^−1^ (c) and (d) showed the aromatic skeleton vibrations. C-H deformation (asymmetric in -CH_3_ and -CH_2_-) was shown in the 1460 cm^−1^ wavelength of lignin (b), (c), and (d). At a wavelength of 1422 cm^−1^, lignin (a), (c), and (d) showed C-H deformation in lignin. C-H stretching of G units was shown from lignin (a) and (b) in the 1283 cm^−1^. The peaks in 1162 cm^−1^ showed by lignin (a), (b), (c), and (d) were typical for H, G, and S units of lignin. In 1110 cm^−1^ peak, lignin (a), (b), (c) and (d) showed aromatic C-H deformation of syringyl units, while in 917 cm^−1^, lignin (a) and (b) showed C-H bending of the syringyl unit’s aromatic ring. The C-H bending of syringyl units was shown in 840 cm^−1^ of lignin (c) and (d). Peaks observed in FTIR spectra and the functionalities are tabulated in Table 1.

Figure 2 shows that the lignin from organosolv extraction of various biomass waste had syringyl and guaicyl groups. They are like a typical lignin standard. Meanwhile, the functional group from soda extraction for various biomass waste did not have the group of syringyl and guaicyl but only had the stretching unit in the aromatic group of lignin. This confirms that the organosolv process can extract lignin while saving most of the original structure of lignin. 

Lignin molecular weight can be indicated by the viscosity of the lignin solution [26]. Lignin precursors have different fractions of lignin-forming monomers so they can produce different molecular weights. The viscosity was measured by a vapor pressure osmometer with NaOH 0.5 N as a solvent. Table 2 displays the viscosity of lignin solutions.

As can be seen in Table 2, lignin, which was extracted using the organosolv process at 150 °C, features the highest value of viscosity compared to the other lignin sources. It can be an indication that the molecular weight of the mangosteen peel is higher compared to the other lignin sources. With respect to the Soda process, the viscosity of lignin obtained from the organosolv method has a higher value. The low viscosity of lignin obtained from the Soda process suggests that the molecular structure of lignin was fragmented into smaller forms [27,28]. Interestingly, using the organosolv extraction process, the structures of the lignin are more or less preserved, as indicated by the high viscosity.

### 2.3. Characteristics of Porous Carbon Produced from Lignin

**Figure 3:**
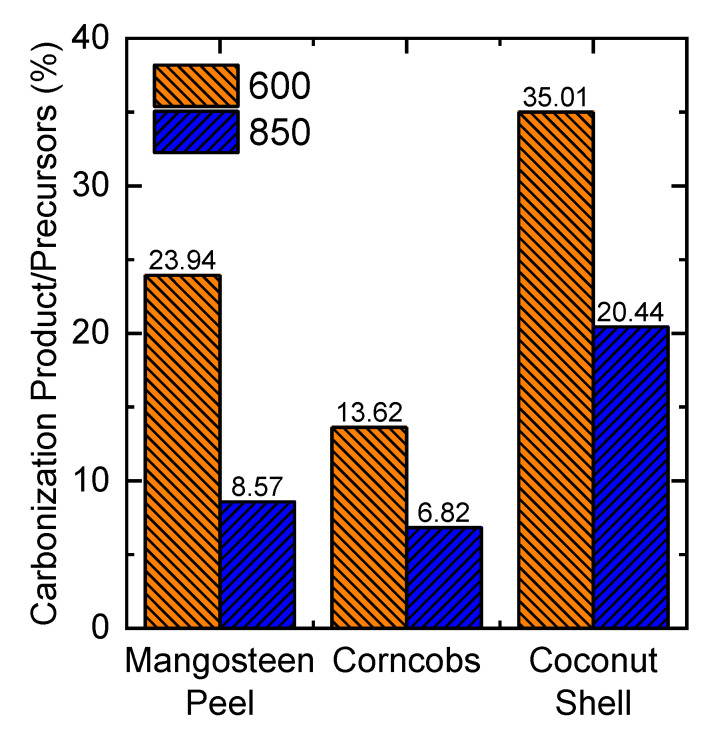
Percentage of product/precursor at carbonization temperatures 600 °C and 850 °C. Data of carbonization yield of mangosteen peel taken from our previous study [18].

The precursors used in porous carbon preparation were lignin extracted from mangosteen peel (MP), corncob (CC), and coconut shell (CS). The temperatures used for the carbonization process were 600 °C and 850 °C. Figure 3 shows that at 600 °C, more carbon products were obtained than at 850 °C for all of biomass waste precursors. The increase of carbonization temperature resulted in decreasing the product. This was because of a thermal degradation process in the molecular structures of lignin. Lignin extracted using the Soda process was also carbonized at 600 °C. The yield was lower than 10%, which is likely due to low thermal degradation of lignin obtained from the Soda process (data not shown, see literature [18]). The carbon produced was labeled in accordance with the type of lignin and carbonization temperature. For instance, LMP-850 means porous carbon produced by pyrolysis of lignin from mangosteen peel at 850 °C.

**Figure 4:**
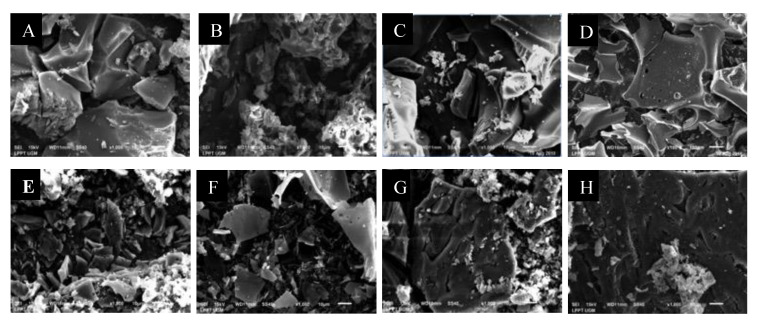
SEM images of carbon fibers: (**a**) LMPT-600; (**b**) LMPT-850; (**c**) LMP-600; (**d**) LMP-850; (**e**) LCC-600; (**f**) LCC-850; (**g**) LCS-600; (**h**) LCS-850. Figure (**c**) and (**d**) taken from our previous study [18], Copyright AIP Publishing, 2020.

Scanning electron microscopy (SEM) analysis was performed to evaluate the details of the microscopic surface structure of the carbon material. The carbon morphology in Figure 4a shows a rigid and ordered pore structure similar to Figure 4c. The LMPT-850 carbon in Figure 4b shows morphology like a sponge with more voids. The SEM result of LMP-850 in Figure 4D shows flake structures. For the LCC-600 and LCC-850 (Figure 4e,f), these carbons possess rigid flakes. For carbon from coconut shell, the LCS-850 in Figure 4h shows flatter surfaces with many voids compared with LCS-600 in Figure 4g Therefore, morphologies of carbon depend on the material precursors and carbonization temperature, which is in agreement with the literature [7]. It is important to note that the morphologies of carbon synthesized by carbonization of lignin (lignin-derived carbon) are very different from the morphologies of porous carbon produced directly from biomass of mangosteen peel [29], corncob [30] and coconut shell [31].

The carbon materials, from the carbonization process for various precursors and carbonization temperatures were characterized by nitrogen adsorption and desorption. From N_2_-sorption isotherms, pore structure, pore size distribution and micropore structure were evaluated. The characterization of pore size distribution was used to evaluate the uniformity of pore sizes formed in the carbonization process. The uniformity of pore size can be seen from a narrow pore size distribution. The quenched solid density functional theory (QSDFT) method was used to evaluate the pore size distribution of produced carbon. According to the International Union of Pure and Applied Chemistry (IUPAC), pores with a size of 2–50 nm are classified as mesoporous while below 2 nm categorized as micropores [32].

**Figure 5:**
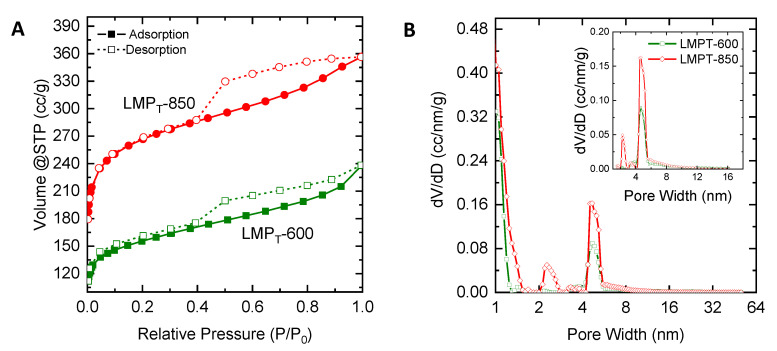
(**A**) Adsorption and desorption of nitrogen in carbon mangosteen peel lignin extracted using organosolv at 150 °C; (**B**) QSDFT-N_2_ pore size distribution of carbon mangosteen peel lignin extracted by using organosolv at 150 °C.

Figure 5A displays the nitrogen adsorption-desorption curves of LMPT for the carbonization temperature of 600 °C and 850 °C. Both carbons show the isotherm of type IV (IUPAC Classification [32]), where the shape of this isotherm curve type becomes more vertical in the range of relative pressure near 1.0. In the specific range, the desorption line does not coincide with the adsorption line, which is called the hysteresis phenomenon. This phenomenon happens because there is a difference in the contact angle of the gas molecules in the pore when adsorption and desorption happen. Condensation in the capillaries of carbon pores is also another reason for this phenomenon [33].

Figure 5B shows the pore size distribution of mangosteen peel lignin extracted using the organosolv process at 150 °C. Both carbons feature a pore size in the range of 1–50 nm, which is in the micropore and mesopore ranges. The pore size distribution for both carbons is broad and diverse. The majority of pores are in the micropore range as indicated by a high fraction of pores below 2 nm. Interestingly, LMPT-600 also features pores in a mesopore range and a narrow pore size distribution with a peak of ca. 5 nm. This peak becomes more intense in LMPT-850, indicating that more mesopores are produced at a higher carbonization temperature. The presence of mesopore is likely associated with a large amount of decomposition of material [34] and a minor fraction of mineral in the lignin [35]. 

**Figure 6:**
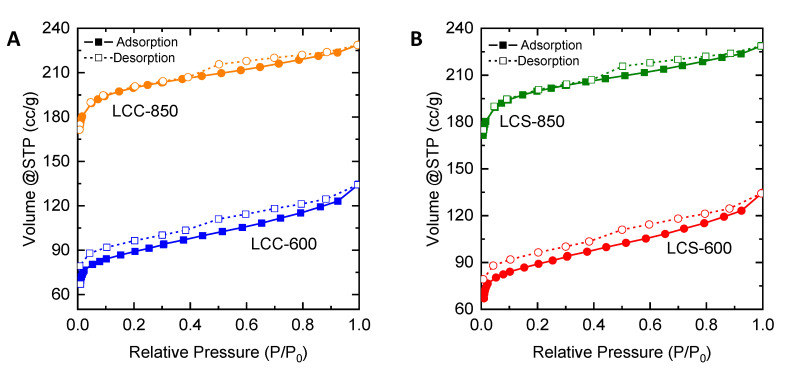
Adsorption and desorption of nitrogen in carbon (**A**) corncob lignin; (**B**) coconut shell lignin.

Figure 6 displays the nitrogen adsorption-desorption curves for carbon produced from lignin of corncob and coconut shell. The LCC-600 in Figure 6A and LCS-600 in Figure 6B show the type II isotherm (IUPAC Classification [32]) while LCC-850 in Figure 6A and LCS-850 in Figure 5B show the type IV isotherm. The change of the isotherm curve is likely caused by effective pore formation during the carbonization process at higher temperature [35].

**Figure 7:**
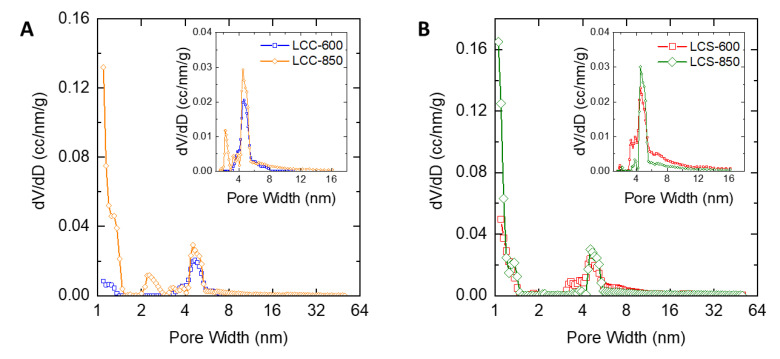
QSDFT-N_2_ pore size distribution of carbon (**A**) corncob lignin; (**B**) coconut shell lignin.

Figure 7A shows the pore size distribution of LCC at the carbonization temperatures of 600 °C and 850 °C while Figure 6B displays the pore size distribution of LCS at the carbonization temperatures of 600 °C and 850 °C. All carbons had a pore size between 1–50 nm, which is in the range of micropores and mesopores, where all the carbons had multimodal or broad pore size distribution.

The isotherm curves of nitrogen adsorption-desorption from Figure 5 and Figure 6 were used for determining the pore structure. The calculation of specific surface area (S_BET_), mesopore specific surface area (S_meso_), pore volume (V), mesopore volume (V_meso_) and average pore diameter (D_avg_) for various precursor are shown in Table 3.

From Table 3, it is shown that LMPT-850 (974 m^2^/g) possesses a higher specific surface area than the LMPT-600 (567 m^2^/g) but both carbons are microporous carbons because of the low percentage of mesopore surface area (<20%). The average diameter for LMPT-850 is 2.04 nm, while LMPT-600 is 2.50 nm. For LMP, the one carbonized at 600 °C features a high percentage of mesopore surface area (41.76%) with an average pore diameter of 3.04 nm, while that with a carbonization temperature of 850 °C shows a lower percentage of mesopore area (7.06%) with an average pore diameter of 1.47 nm. LMP-850 carbon is a micropore carbon, which gives a higher specific surface area (595 m^2^/g) than LMP-600 carbon (205 m^2^/g). All LCC and LCS carbons show similar carbon pore characteristics with a low percentage of mesopore surface area less than 20%. Both LCC-850 (820 m^2^/g) and LCS-850 (781 m^2^/g) feature a higher specific surface area than LCC-600 (349 m^2^/g) and LCS-600 (333 m^2^/g) with average diameters in the range of 1.4–1.9 nm. 

In this work, porous carbon produced by carbonization of lignin had specific surface area in the range of 205–974 m^2^/g depending on lignin material and carbonization temperature. A higher temperature was favorable to obtain a high specific surface area. The highest specific surface area was 974 m^2^/g, which can compete with commercially available porous carbon [36] and lignin-derived carbon as in the literature [37]. 

## 3. Materials and Methods 

### 3.1. Materials

The biomass precursors used in this experiment were obtained from different places. Mangosteen peel, which had 180–355 μm particle size, was obtained from Bina Agro Mandiri, Bantul, D.I. Yogyakarta, Indonesia. The corncob was obtained from old pearl corn in Sripendowo Village, South Lampung, Indonesia. The coconut shell with dark brown color and size of 80 mesh was obtained from Ruko Taman Niaga, Semarang, Indonesia. The solvent for lignin extraction was ethanol (96%, Sigma Aldrich, Singapore). Sulfuric acid (98%, Sigma Aldrich, Singapore) was employed for lignin precipitation.

### 3.2. Methods

Extraction using the organosolv process at low temperature was carried out based on the method described in the literature [18,38]. Firstly, mangosteen peel was poured in ethanol (60%) in a three-neck flask. The ratio of solid:liquid was set to 1:8 (*w*/*v*). The extraction process was carried out at 70 °C for 4 h. After that, the black liquor obtained was separated from the solid phase using filtration. Precipitation of lignin was carried out by adding sulfuric acid 1 N until the pH was 2. The lignin obtained was then taken from solution by centrifugation and dried in an oven overnight. Similar procedures of the organosolv process at low temperature were carried out for the corncob and coconut shell as raw materials of biomass. The lignin produced was labeled depending on the type of biomass i.e., LMP (lignin from mangosteen peel), LCC (lignin from corncob) and LCS (lignin from coconut shell). The extraction process at high temperatures was carried out in an autoclave reactor at a temperature of 150 °C and a pressure of 10 bar under nitrogen pressure. For extraction using the Soda process, a similar procedure was employed but the solvent was 10% sodium hydroxide (see the literature [18]). The properties of lignin were investigated by Fourier-transform infrared (FTIR) spectroscopy (Thermo Nicolet iS10 by Thermo Fisher Scientific, MA, USA) and lignin solution viscosity (DV-E Viscometer by Brookfield, Toronto, Canada). 

Conversion of lignin to carbon was conducted by a carbonization process in a furnace at 600 °C and 850 °C under a flow of nitrogen. The detailed procedure is available elsewhere [38]. The carbon produced was labeled in accordance with the type of lignin after carbonization temperature. For instance, LMP-600 means porous carbon produced by pyrolysis of lignin from mangosteen peel at 600 °C. The pore structure of carbon was characterized by N_2_-sorption analysis (NOVA 2000 by Quantachrome Instruments, Boynton Beach, FL, USA) and scanning electron microscopy, SEM (JSM 6510 LA by JEOL, Tokyo, Japan).

## 4. Conclusions

Lignin was successfully extracted by the organosolv process. The yield of lignin depended on the types of biomass and extraction conditions. A higher extraction temperature was more favorable to obtain a greater amount of lignin. The FTIR spectra of lignin showed peaks of syringyl and guaiacyl units, which are typical features for a lignin standard. When lignin was converted to porous carbon, the temperature of pyrolysis significantly affected the yield and properties of the porous carbon produced. Pore structure analysis displayed that carbon from lignin of mangosteen peel, corncob, and coconut shell synthesized at 850 °C exhibited a high surface area of >550 m^2^/g. The highest surface area, up to 974 m^2^/g, was obtained in this work. The results showed that lignin-derived carbon is promising and could be beneficial for porous carbon applications.

## Figures and Tables

**Table 1:** Peaks Observed in FTIR Spectra.

Sample	Wavenumber (cm^−1^)	Functionality
LMP; LCC; LCS; LMPT	1600	C=C stretching of the aromatic ring
LCS; LMPT	1510	Aromatic skeleton vibrations
LCC; LCS; LMPT	1460	C-H deformation (asymmetric in -CH_3_ and -CH_2_-)
LMP; LCS; LMPT	1422	C-H deformation in lignin
LMP; LMPT	1283	C-H stretching of G units
LMP; LCC; LCS; LMPT	1162	H, G, and S units of lignin
LMP; LCC; LCS; LMPT	1110	Aromatic C-H deformation of syringyl units
LMP; LMPT	917	C-H bending of syringyl units, aromatic ring
LCS; LMPT	840	C-H bending of syringyl units

**Table 2:** Viscosity of lignin.

Lignin Source	Viscosity (cP)	
	Organosolv Process	Soda Process
Mangosteen peel	1.43/1.97 (at 150 °C)	1.23
Corn cob	1.63	1.17
Coconut shell	1.53	1.20

**Table 3:** Structural pore properties of carbon. Data of LMP-600 and LMP-850 taken from our previous study [18].

Characteristics	LMP_T_-600	LMP_T_-850	LMP-600	LMP-850	LCC-600	LCC-850	LCS-600	LCS-850
Surface area (S_BET_), m^2^/g	567	974	205	595	349	820	333	781
Mesopore area (S_meso_), m^2^/g	110	153	85	42	28	59	58	50
%S_meso_	19.5	15.67	41.76	7.06	8.06	7.18	17.55	6.34
Total pore volume, cm^3^/g	0.37	0.55	0.21	0.28	0.16	0.38	0.21	0.35
Mesopore volume (V_meso_), cm^3^/g	0.18	0.22	0.16	0.07	0.03	0.09	0.09	0.07
%V_meso_	48.4	40.21	72.40	23.8	17.03	22.61	45.27	20.64
Mean pore diameter, nm	2.50	2.04	3.04	1.47	1.38	1.64	1.88	1.45
